# Brazilian Amazon indigenous territories under deforestation pressure

**DOI:** 10.1038/s41598-023-32746-7

**Published:** 2023-04-10

**Authors:** Celso H. L. Silva-Junior, Fabrício B. Silva, Barbara Maisonnave Arisi, Guilherme Mataveli, Ana C. M. Pessôa, Nathália S. Carvalho, João B. C. Reis, Admo R. Silva Júnior, Nathalia A. C. S. Motta, Paulo Vinícius Moreira e Silva, Francarlos Diniz Ribeiro, Juliana Siqueira-Gay, Ane Alencar, Sassan Saatchi, Luiz E. O. C. Aragão, Liana O. Anderson, Maycon Melo

**Affiliations:** 1 Institute of Environment and Sustainability, University of California Los Angeles - UCLA, Los Angeles, CA USA; 2 Jet Propulsion Laboratory - JPL, California Institute of Technology, Pasadena, CA USA; 3 Programa de Pós-Graduação em Biodiversidade e Conservação, Universidade Federal do Maranhão - UFMA, São Luís, Brazil; 4 Programa de Pós-Graduação em Meio Ambiente, Universidade Ceuma - UNICEUMA, São Luís, MA Brazil; 5 Vrije Universiteit Amsterdam, Amsterdam, The Netherlands; 6 National Institute for Space Research - INPE, São José Dos Campos, SP Brazil; 7 National Center for Monitoring and Early Warning of Natural Disasters - Cemaden, São José Dos Campos, SP Brazil; 8 Instituto de Pesquisa Ambiental da Amazônia - IPAM, Brasília, Brazil; 9 Instituto Escolhas, São Paulo, Brazil; 10 University of Exeter, Exeter, UK

**Keywords:** Climate sciences, Environmental sciences, Environmental social sciences

## Abstract

Studies showed that Brazilian Amazon indigenous territories (ITs) are efficient models for preserving forests by reducing deforestation, fires, and related carbon emissions. Considering the importance of ITs for conserving socio-environmental and cultural diversity and the recent climb in the Brazilian Amazon deforestation, we used official remote sensing datasets to analyze deforestation inside and outside indigenous territories within Brazil's Amazon biome during the 2013–2021 period. Deforestation has increased by 129% inside ITs since 2013, followed by an increase in illegal mining areas. In 2019–2021, deforestation was 195% higher and 30% farther from the borders towards the interior of indigenous territories than in previous years (2013–2018). Furthermore, about 59% of carbon dioxide (CO_2_) emissions within ITs in 2013–2021 (96 million tons) occurred in the last three years of analyzed years, revealing the magnitude of increasing deforestation to climate impacts. Therefore, curbing deforestation in indigenous territories must be a priority for the Brazilian government to secure these peoples' land rights, ensure the forests' protection and regulate the global climate.

## Introduction

About 700 thousand indigenous people live in Indigenous Territories (ITs) in Brazil^1^, and over half of ITs are within the Brazilian Legal Amazon^2^. The ITs host more than one million^3^ square kilometers of rainforests that contribute to climate regulation^4,5^, store carbon^6,7^, and are home to unique biodiversity^8^ and sociocultural diversity of indigenous peoples^9^. Going beyond the passive benefits of a conserved area, protected areas, including ITs, can still act as shields against deforestation and forest degradation^10–13^. By ensuring land rights for the indigenous people, these territories in the Brazilian Amazon could decrease deforestation by up to 66%^14^. Therefore, the ITs are not only essential for maintaining indigenous ways of life, having a key role in ensuring the human rights of these peoples, but they are also a cost-effective strategy for conservation^10,11,14^. Furthermore, global protected areas, including ITs, could improve carbon sequestration^15^, highlighting the positive externality for the whole world regarding climate change mitigation these areas could impose^14^.

ITs are crucial in reducing carbon emissions and mitigating global climate change^16^. Keeping these forests intact and protected makes it possible to avoid releasing large amounts of CO_2_ into the atmosphere, reducing greenhouse gas emissions. The maintenance and protection of ITs are necessary because deforestation and forest degradation in these areas can release large amounts of CO_2_ into the atmosphere^17,18^. In addition, the indigenous communities that inhabit these protected areas have land use practices that are often sustainable and in harmony with the environment, such as agroforestry systems, fishing, and subsistence hunting. These practices can contribute to the conservation of the forest and the reduction of carbon emissions^16^.

Among tropical countries, in 2012, Brazil achieved an important achievement, reducing deforestation rates in the Amazon by 84% (4571 km^2^) from the historical peak of 2004^19^ (27,772 km^2^). This reduction resulted from multiple government initiatives, especially the Action Plan for the Prevention and Control of Deforestation in the Legal Amazon (PPCDAm)^20^ and international pressure, such as the soy and beef moratoria^21^. However, there is a scenario of gradual deforestation growth in the Brazilian Amazon since 2013^19^, and a set of environmental setbacks^19,22–25^ (e.g., weakening of environmental agencies and relaxation of environmental policies) in the last few years took 2020 to the highest deforestation rate of the decade^19^ (10,851 km^2^). Surprisingly, in 2021, the deforestation rate surpassed 2020 by 20%, reaching 13,038 km^2^ of clear-cut forests^26^. The setback in the environmental agenda has also affected indigenous people's policies^27–29^. This scenario has imperiled the ITs^30,31^, compromising their environmental potential and role in the survival of indigenous life, culture, and knowledge.

Thus, to accurately diagnose deforestation dynamics in the Brazilian Amazon ITs, we used two official remote sensing datasets and statistical analyses to perform a space–time assessment of deforestation in indigenous territories within the Brazilian Amazon biome between 2013 and 2021; we adopted this time window because it was considered a period of a new deforestation climb in the Brazilian Amazon^19^, mainly in the 2019–2021 period when there was an intensification of environmental setbacks in the region^23,32^. Considering the importance of ITs for conserving socio-environmental and cultural diversity and the recent climb in the Brazilian Amazon deforestation, we provide to answer the following questions: (1) Was there a significant trend in deforestation rates inside and outside ILs between 2013 and 2021? (2) Within the ILs, was deforestation significantly higher in the 2019–2021 period? (3) In the 2019–2021 period, did deforestation advance to the interior of the ILs? (4) How much was carbon dioxide (CO_2_) emitted from deforestation between 2013 and 2021?

## Results and discussion

**Figure 1:**
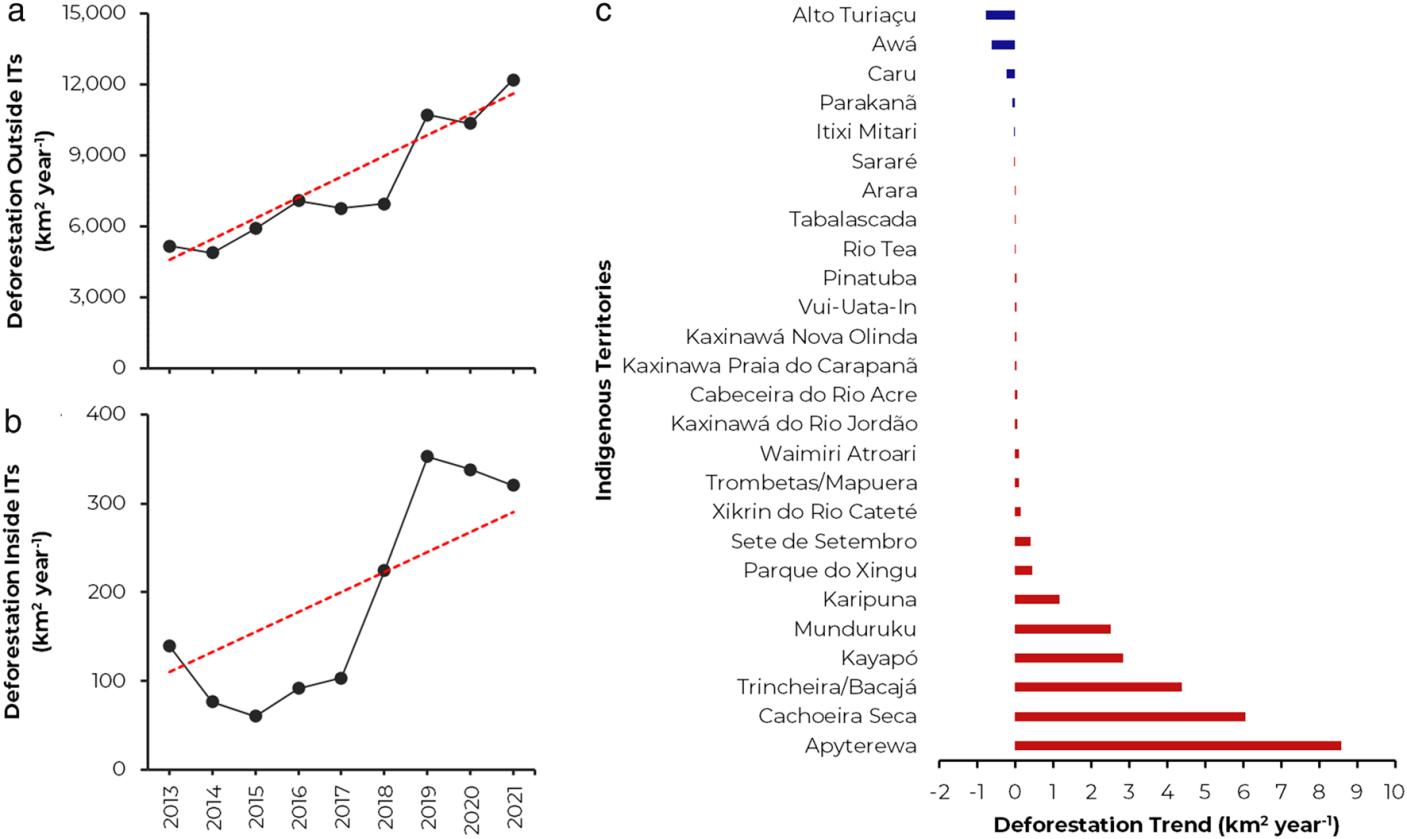
(**a**) Annual deforestation outside indigenous territories between 2013 and 2021 in the Brazilian Amazon biome. (**b**) Annual deforestation inside indigenous territories between 2013 and 2021 in the Brazilian Amazon biome. (**c**) Indigenous territories with a significant deforestation trend (*p* < 0.05) between 2013 and 2021. The figure was elaborated by Celso H. L. Silva-Junior using the software Microsoft Office Excel (https://www.microsoft.com/pt-br/microsoft-365).

We found that outside ITs, deforestation increased significantly at a rate of 900 km^2^ year^-1^ (*p* < 0.05 and MK = 28; MK is the Mann–Kendall test statistic), or an increase of 137% in the 2013–2021 period (Fig. 1a). Following the same pace, inside of the 232 analyzed ITs, deforestation increased significantly at a rate of 35 km^2^ year^-1^ (*p* < 0.05 and MK = 20), or an increase of 129% in the same period (Fig. 1b). Between 2013 and 2021, deforestation within TIs reached a total area of 1708 km^2^ or 2.38% of all deforestation in the Brazilian Amazon for the same period; per period, we found that between 2013 and 2018, 696 km^2^ of forest were lost or 1.86% and between 2019 and 2021 1,012 km^2^ or 2.95% of all deforestation in the Brazilian Amazon in the same time window.

**Figure 2:**
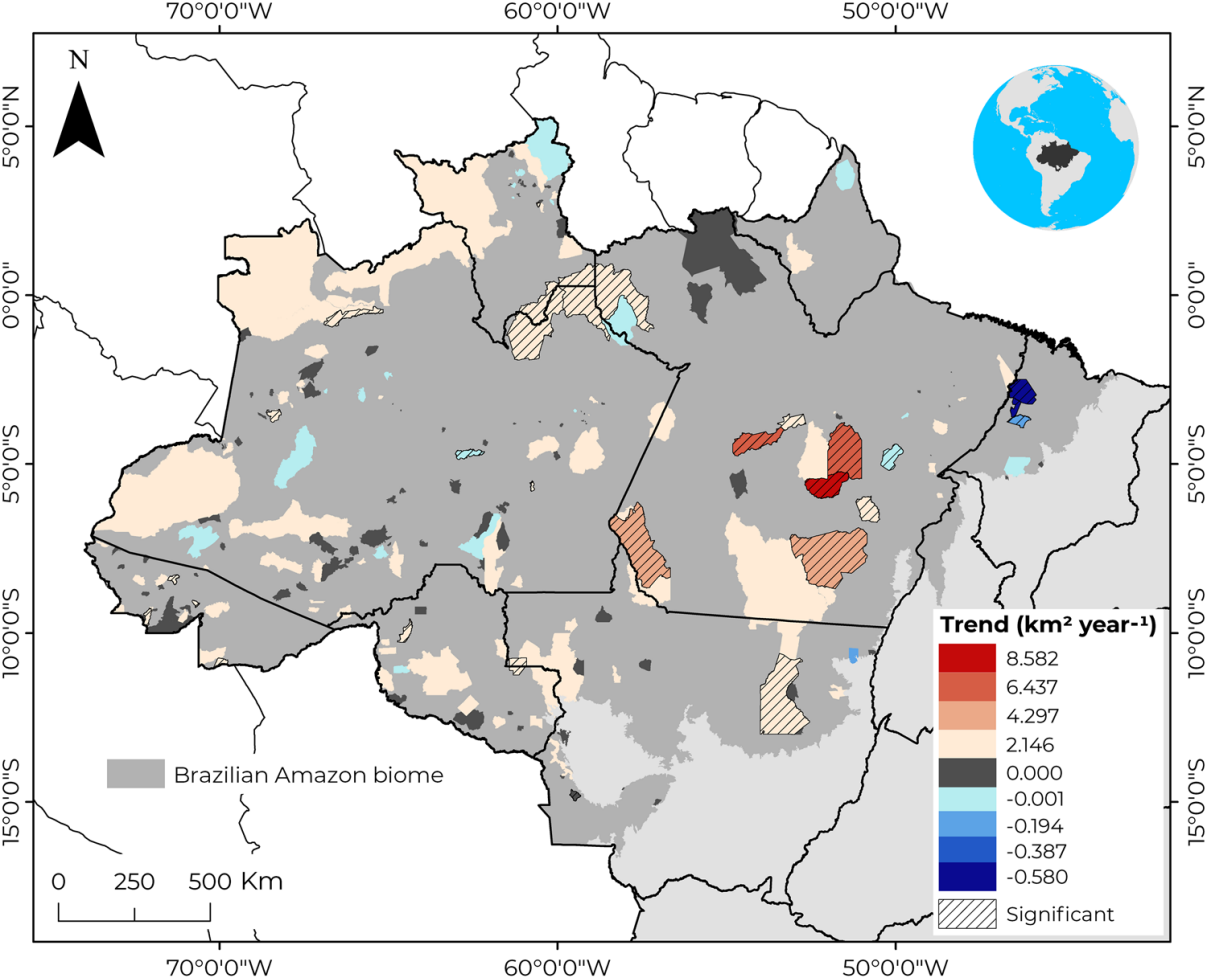
Space–time deforestation trends within the Indigenous Territories of the Brazilian Amazon biome between 2013 and 2021. On the map, indigenous territories with an increasing trend in deforestation are filled in shades of red, while those with a decreasing trend are filled in blue. Indigenous territories filled in grey had no trend in the analyzed period. An interactive map from the Socio-environmental Institute—ISA with the location and basic information of each Brazilian indigenous territory can be accessed at: https://terrasindigenas.org.br/en. The map was elaborated by Celso H. L. Silva-Junior using the software ArcGis 10.8 (https://www.esri.com).

Our local trend analysis (Figs. 1c and 2) of each 232 ITs showed that 42% of analyzed ITs had an increased deforestation rate, although, in 20 of them, the trend was significant (*p* < 0.05); among the ITs with a significant increasing trend, *Arara* in Pará had the lowest rate (0.02 km^2^ year^-1^), while *Apyterewa* in the same state had the highest rate (8.58 km^2^ year^-1^). On the other hand, 11% of the ITs tended to reduce deforestation, with five being significant (*p* < 0.05); among the ITs with a significant decreasing trend, *Alto Turiaçu* in Maranhão had the lowest rate (− 0.77 km^2^ year^-1^), while *Itixi Mitari* in Amazonas had the highest rate (− 0.01 km^2^ year^-1^). Another 47% of the ITs did not show a trend in the analyzed period (Table S1).

These findings suggest that the increase in deforestation inside ITs (Fig. 1), induced by recent environmental setbacks^23,32^, may reverse the vital role of these protected areas in combating climate change^33^ by avoiding deforestation. Previous work that analyzed past periods found that Brazilian Amazon protected areas are efficient in preventing deforestation^10,34–38^; however, in the most recent period, we have found evidence to argue that the role of ITs is being counteracted by the weakening of governance, decrease in the protection and rights of indigenous people^19,22–25^. In addition, the increased deforestation in legally protected areas undertaking climate agreements and the achievement results in REDD + (Reducing Emissions from Deforestation and Forest Degradation) programs.

Within ITs, deforestation led to the emission of 96 Tg CO_2_ (millions of tons of CO_2_) into the atmosphere between 2013 and 2021 (Table 1); this amount is 1.83 times greater than the annual average of Brazilian emissions by the industrial process sector (52 ± 23 Tg CO_2_ year^-1^; 1970–2021 period)^39,40^. About 59% of these emissions (57 Tg CO_2_) were concentrated between 2019 and 2021 (average of 19 Tg CO_2_ year^-1^), while the remaining 41% (39 Tg CO_2_) occurred in the 2013–2018 period (average of 6 Tg CO_2_ year^-1^). Thus, on average, in 2019–2021, CO_2_ emissions within ITs were almost three times higher than in the 2013–2018 period.

**Table 1:** Carbon dioxide (CO_2_) emissions within analyzed ITs between 2013 and 2021.

Year	Emission (Tg CO_2_ year^-1^)
2013	7.83
2014	4.28
2015	3.38
2016	5.12
2017	5.78
2018	12.57
2019	19.76
2020	18.95
2021	17.95
Total	95.63

Although economic activities are not allowed inside ITs, these territories have recently been threatened by illegal mining^41,42^. Our findings showed that inside the ITs, deforestation notice^26^ classified as mining areas increased from 1% in 2016 to 14% in 2021. However, the most worrying point is that only in half of the 2022 year, mining is already equivalent to 19% of all deforestation notices^26^ in the analyzed ITs. Recent environmental setbacks, which include political speech, can explain the increased illegal mining activity in ITs^23,32^, besides the possibility of regulating commercial mining in these territories^41,43,44^. Combined with setbacks, the rise in the gold price in recent years has increased pressure on these protected areas; in the 2019–2021 period, the gold price increased more than 30%, reaching up to $65 g^−1^, the highest value of the last ten years (https://goldprice.org). In addition to the environmental impact, the anthropic advance on indigenous territories also serves as a vector of diseases, such as malaria, associated with illegal mining activities, which may decimate indigenous peoples along with their culture and knowledge^45–47^.

**Figure 3:**
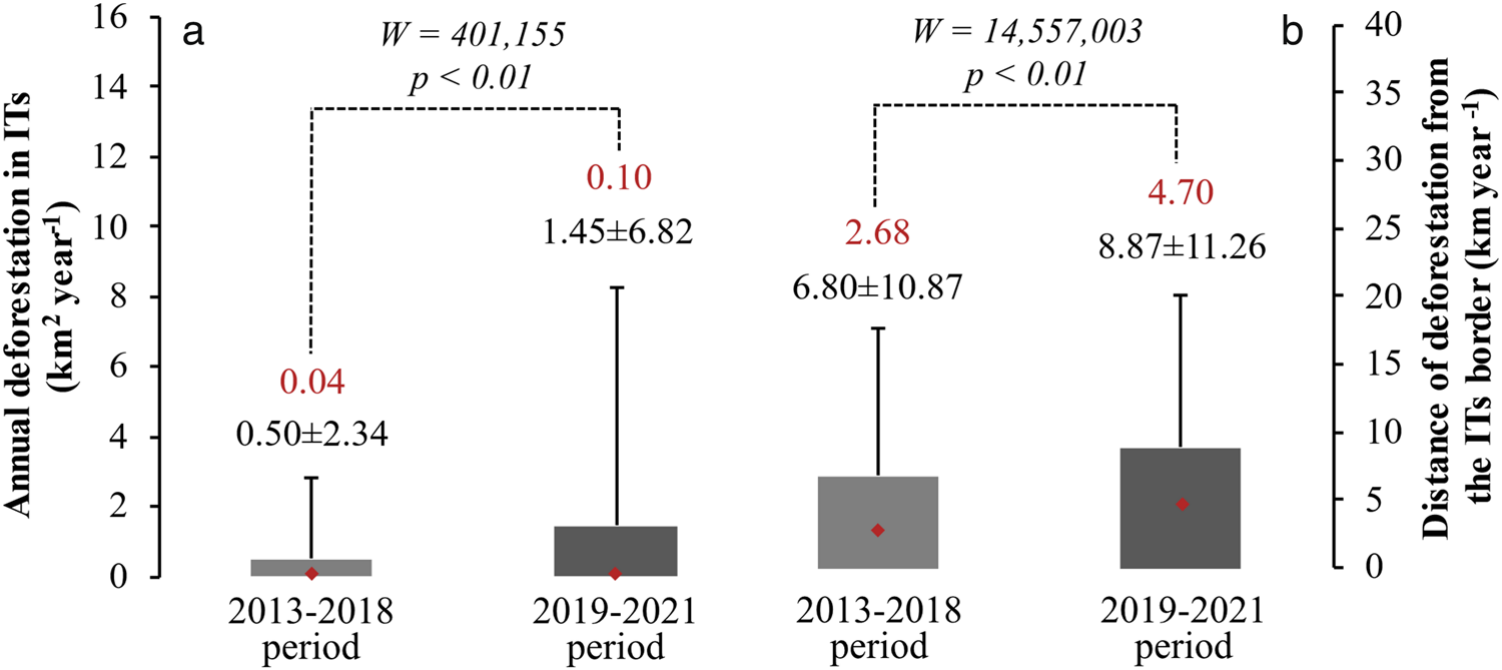
(**a**) Average deforestation within each indigenous land before (2013–2018) and during the current environmental setback intensification period (2019–2021). (**b**) Mean distance from deforestation polygons within indigenous lands to borders before (2013–2018) and during the current environmental setback intensification period (2019–2021). The numbers in red represent medians. The numbers after the plus/minus signal represent the standard deviation. The figure was elaborated by Celso H. L. Silva-Junior using the software Microsoft Office Excel (https://www.microsoft.com/pt-br/microsoft-365).

Comparing deforestation within the ITs during the 2013–2018 and 2019–2021 period, we find that, on average, deforestation has increased substantially from 0.50 to 1.45 km^2^ year^−1^, or 195% (Fig. 3a). Furthermore, the distance of deforested areas from ITs borders also increased significantly from an average of 6.80 to 8.87 km year^−1^, or 30% during the analyzed periods (Fig. 3b). This reveals an increasing threat to these areas, pressuring for more occupation inside these territories.

Although in magnitude, deforestation is greater outside than inside ITs; since 2013, deforestation has increased within ITs, which was significantly higher in the last three years, and caused mainly by illegal mining^44^. Our findings indicate that the setback of environmental regulations^22–25,48^ and the weakening of policies protecting indigenous people's lands in recent years^27–29^ have impeded the ability to prevent deforestation inside ITs^10,49^. This has led to increasing conflicts in the region and further vulnerability of indigenous people to threats of degradation of their habitat and livelihood^30,50,51^ and exposing them to COVID-19, which has an infection rate in these populations 84% higher than the Brazilian population^52^.

The recent increase in deforestation in the Brazilian Amazon is a severe threat to decade-long efforts of curbing climate change. Indigenous communities have been the most effective defenders and consistent guardians of the Amazon rainforests^33^. With their land threatened by illegal mining^41,43,44^ and land grabbing^53^, these communities' management systems and environmental services over millennia^54^ will be compromised substantially. Most ITs have full legal property rights under presidential homologation, and the government is responsible under the constitution to protect and respect the rights of these territories^14^. Unfortunately, the past government (in power between 2019 and 2022) ignored these responsibilities, allowing the indigenous lands to be exploited by illegal mining and land grabbing, inflating the conflicts in the region^55,56^.

While remote sensing data are valuable for studying the Earth's surface^57^, including advancing deforestation within indigenous territories, its limitations must be considered. We used two official deforestation mapping datasets, PRODES (Brazilian Amazon Deforestation Monitoring Program) and DETER (Brazilian Amazon Near Real-Time Deforestation Detection System). Both deforestation monitoring systems can be affected by the high cloud cover^58,59^ in the Amazon—mainly in the northern region—leading to the underestimation of forest loss mapping. Furthermore, the minimum mapped area (3–6.25 hectares) in both systems may lead to underestimating small-scale deforestation. Another is related to CO_2_ estimates; however, we used a method widely accepted in the literature^60^, reproducible and which produces results that can be compared with others in the literature. In addition, for our CO_2_ model, we provided uncertainty analysis (95% confidence interval) as reported in Fig. S1 of the supplementary material.

Thus, although the estimate of deforestation magnitude used here is conservative, its trends are not affected and are consistent with other datasets reported in the literature^61–63^. Improvements that increase monitoring frequency and scale may help overcome deforestation mapping limitations. Furthermore, other datasets and CO_2_ emission estimation models may be tested in the future.

## Conclusion

Here we find a recent increase in deforestation within ITs that coincided with a decrease in the protection and rights of indigenous people. We can now answer that: (1) there was a significant trend towards an increase in the area of forests deforested inside and outside the ITs between 2013 and 2021, with an important intensification in the 2019–2021 period, (2) within the ITs, deforestation increased significantly by 195% between 2019 and 2021, (3) in the 2019–2021 period, deforestation advanced 30% from the borders to the interior of the ITs and (4) about 96 million tons of CO_2_ were emitted due deforestation between 2013 and 2021 in the analyzed ITs.

Based on our findings, we believe these territories will remain vulnerable without policy action to combat illegal deforestation in these territories and enforce the constitutional rights of indigenous peoples to protect their lands. For this, we present below a set of recommendations that can be adopted to achieve this objective by federal and state decision-makers:

1. Strengthening of the *Fundação Nacional do Índio* (Brazil's National Indian Foundation), the federal agency responsible for securing indigenous rights, including new ITs demarcation, through more funding and hiring new staff; ensuring the involvement of indigenous communities in decision-making processes and supporting their efforts to protect their lands.
2. Revocation of laws and norms that led to environmental setbacks. In the short term, it would reverse the weakening of local governance and curb further deforestation led by illegal mining and land grabbing.
3. Strengthening of enforcement institutions that operate inside and outside indigenous territories, prioritizing the ITs with a significant trend of increased deforestation, was found in our analysis (Table S1).
4. Creation of a buffer zone of 10 km between ITs and mining exploration areas or high-impact projects^64^. In addition to canceling all CARs (Rural Environmental Registry) within the ILs, including the impossibility of new registrations in these territories^64^.
5. Support initiatives that promote agriculture, forestry, and other sustainable land-use practices, including ecosystem restoration projects to reduce the pressure of deforestation on surrounding ITs.
6. Strengthening existing remote sensing monitoring and investments in developing new systems with improvements in frequency and scale. In addition, forest degradation and non-forest native vegetation must also be monitored.

Finally, we highlight the need for future work considering ITs outside the Brazilian Amazon and deforestation in non-forest native vegetation. For example, the Cerrado, the second largest biome in Brazil, has a predominance of non-forest native vegetation and increasing deforestation rates^65^.

## Methods

### Remote sensing deforestation datasets

We analyzed two official remote sensing-based deforestation datasets throughout the Brazilian Amazon Biome between 2013 and 2021. This time window was adopted as it was considered a period of a new deforestation climb in the Brazilian Amazon^19^. Therefore, we adopted two analysis periods, before (2013–2018) and during the current environmental setback intensification period (2019–2021)^23,32^. Each dataset is described below.

#### Brazilian Amazon deforestation monitoring program (PRODES)

PRODES was created in 1988 and has since provided the official annual deforestation rates of the Brazilian Legal Amazon. The system annually identifies clear-cut deforestation of old-growth forests. Once identified as deforested, such an area is not revisited in the subsequent year mapped. The monitoring system uses medium spatial resolution images (20–30 m) in a combination that seeks to minimize cloud cover interference and ensure interoperability criteria. Landsat-5 satellite images have historically been the most used, but CBERS (Sino Brazilian remote sensing program) satellite images have been widely used. PRODES also used the Indian IRS-1 satellite and UK-DMC2 United Kingdom satellite.

The reference year comprises August 1st of the prior year to July 31st of the reported year. The annual rate is first estimated using a subset of images from the deforestation hotspots, and the result is published in December of each year. Subsequently, the analysis of the entire dataset, corresponding to the consolidated data, is made available in the first half of the following year. Currently, the system uses the Landsat-8, CBERS-4, and IRS-2 images. PRODES's method encompasses the visual interpretation of images by a team of experts with extensive experience.

The PRODES dataset was obtained in vector format directly from: http://terrabrasilis.dpi.inpe.br/en/download-2.

#### Brazilian Amazon near real-time deforestation detection system (DETER)

DETER was developed in 2004 to support the environmental enforcement of the Brazilian Amazon, generating daily notices of deforestation^66^. Until 2015, this system used satellite images from the Moderate Resolution Imaging Spectroradiometer (MODIS) sensor with 250-m spatial resolution. Currently, DETER uses images from the Wide Field Imager (WFI) and Advanced Wide Field Sensor (AWIFS) sensors, with 64 and 56-m spatial resolution, respectively^67^.

The improved spatial resolution from 2006 allows the monitoring to discriminate alerts between deforestation (including mining areas) and forest degradation. These alerts are sent daily to the Brazilian Institute of Environment and Renewable Natural Resources (IBAMA), the country's environmental enforcement agency in the Brazilian Amazon^67^In addition, environmental agencies of Brazilian states can also access daily information. DETER's method encompasses the visual interpretation of images by a team of experts with extensive experience.

The DETER dataset was obtained in vector format directly from: http://terrabrasilis.dpi.inpe.br/en/download-2.

### Processing deforestation datasets

At this stage, all procedures were performed using ArcGIS^®^ 10.8 software (Environmental Systems Research Institute—ESRI). For area and distance calculations, we consider a plane coordinate system under the Earth surface representation model (Datum) "South America Albers Equal Area Conic (ESRI:102,033)".

Here we adopt the Brazilian Amazon Biome delimitation^68^ 292 legally approved indigenous territories^69^. However, after overlapping the deforestation polygons for the 2013–2021 period, only 232 (about 79%) were considered to have deforestation within their territorial domains.

First, we calculated the area in square kilometers of PRODES deforestation outside (including protected areas) and inside all the analyzed ITs annually. Then we calculated the area of deforestation within each indigenous land each year. Finally, we calculated the distance from the centroid of each deforestation polygon to the nearest indigenous land border.

From the DETER deforestation notices, the mining contribution was calculated in all analyzed ITs for 2016, 2021 and 2022 (January to June).

### Statistical analyses

Here we used nonparametric statistical approaches that do not assume that the data has a known distribution and are lowly sensitive to discrepant values. All analyses were performed using the R statistical software^70^ (version 3.4.4; https://www.r-project.org). In all analyses, we adopted a significance level of 95% (*p* < 0.05).

To analyze the significance and magnitude of temporal deforestation trends inside and outside (all deforestation out of the ITs domain) indigenous territories, we used Mann–Kendall test^71,72^, and Sen's Slope estimator^73^, respectively. For both methods, we used the "wq" R package^74^.

The Mann–Kendall test is an approach that tests whether the observations of a given time series (*x*_1_, *x*_2_, *x*_3,_ …, *x*_n_) are independent and identically distributed. For this, the hypotheses considered are H_0_, which states that observations are independent and identically distributed (there is no trend), and H_1_, which states that observations have a monotonic trend in time (there is a trend). About H_0_, the *S* statistic is given by Eq. (1):

1 $$ S = \mathop \sum \limits_{k = 1}^{n - 1} \mathop \sum \limits_{j = k + 1}^{n} sign\left( {x_{j} - x_{k} } \right) $$

where *n* is the number of points in the series, *x* represents the measurements in time; *i* and *j* are time indices, with *i* ≠ *j*, and sign [Eq. (2)] is defined as:

2 $$ sign\left( x \right) = \left\{ {\begin{array}{*{20}l} {1,\;\;{ }\;\;{\text{if}}\, x > 0} \hfill \\ {0,\;\;\;\; {\text{if}}\, x = 0} \hfill \\ { - 1,\;\;\;\;{\text{ if}}\, x < 0} \hfill \\ \end{array} } \right. $$

The positive values of *S* [Eq. (1)] indicate positive trends over time, and negative values indicate a negative trend. Furthermore, it is necessary to calculate the probability associated with *S* and the sample size *n* to define the significance of the trends. Then, for *n* > 10, a normal approximation for the Mann–Kendall test is considered. Thus, the variance of *S* can be given by Eq. (3):

3 $$ Var\left( S \right) = \frac{{n\left( {n - 1} \right)\left( {2n - 1} \right)\sum\nolimits_{j = 1}^{p} {t_{j} \left( {t_{j} - 1} \right)\left( {2t_{j} + 5} \right)} }}{n} $$

where *p* is the number of groups with equal values in the time series, and *t*_*j*_ is the number of data with similar values in each group *j*. If *S* is normally distributed, with zero mean and variance given by *Var(S)*, it is possible to test whether a positive or negative trend significantly differs from zero. For *S* to be significant and different from zero, H_0_ should be rejected considering the level of significance adopted, pointing to the existence of a trend in the time series, thus accepting H_1_.

After identifying trends in the time series, it is also important to estimate the magnitude. In most of the methods used for this purpose, the normality of the data is a prerequisite, being highly sensitive to outliers. To resolve this limitation, a non-parametric and robust method was developed by Sen^75^ to estimate the magnitude of trends over time series. The Sen statistic is given by the median of the slopes of each pair of points in the data set^75^. To calculate the Sen's Slope Estimator (*Q*), the data should be ranked in ascending order as a function of time, and then Eq. (4) is applied.

4 $$ Q = Median\left\{ {\left[ {\left( {\frac{{x_{i} - x_{j} }}{i - j}} \right)_{j = 1}^{j = n - 1} } \right]_{i = j + 1}^{i = n} } \right\} $$

where *x*_*i*_ and *x*_*j*_ are pairs at given times *i* and *j* (*j* > *i*), respectively.

In addition, to compare the annual deforestation and annual deforestation distance from borders within indigenous territories before and during the current environmental setback intensification period, we used the Wilcoxon test^76^. The Wilcoxon test is equivalent to the student's t-test, which compares two independent groups to test the hypothesis that they have the same median. In addition, we used Fisher's least significant difference criterion with Bonferroni adjustment methods correction^77^. For this method, we used the "*agricolae*" R package^78^.

### Carbon dioxide (CO_2_) emissions from deforestation

In this stage, we used the statistical software Excel^®^ (Microsoft Corporation) for tabulation and linear regression analysis. To build the linear regression model, we used the Data Analysis Tool from the software Excel®.

To estimate the gross CO_2_ emissions due to deforestation, we adopted the approach developed by Aragão et al. (2018)^60^. We regressed the reported deforestation gross CO_2_ emissions from Brazil's Amazon Forest Reference Emission Level (FREL-C)^79^ for the 2008–2015 period (Table 1 from FREL-C document^79^) against PRODES deforestation increment area (within Brazilian Amazon biome) resulting in Eq. (5) (n = 8, R^2^ = 0.99, F = 1255, *p* < 0.01).

5 $$ D_{{CO_{2} }} = 0.056 \left( { \pm 0.004} \right) \cdot D_{Increment} $$

where $D_{{CO_{2} }}$ is the CO_2_ emission from deforestation given in Tg and $D_{Increment}$ the deforestation area given in km^2^. The number in parentheses is the 95% confidence interval for the equation parameter. The chart of the regression line, including the confidence interval and the raw values used (deforestation increment and CO_2_ emissions) are shown in Fig. S1.

## Supplementary Information

Supplementary Information.

## Data Availability

PRODES and DETER datasets are available at: http://terrabrasilis.dpi.inpe.br/en/download-2. In addition, all codes for the data analysis are available from the corresponding author upon reasonable request.
