# Supplementary material for: Brazilian Amazon indigenous territories under deforestation pressure
Source: Sci Rep. 2023 Apr 10;13:5851. doi: 10.1038/s41598-023-32746-7 (PMC10085996; doi:10.1038/s41598-023-32746-7)
Supplement: Supplementary file 1 — Supplementary Information. [file 41598_2023_32746_MOESM1_ESM.pdf]

Supplementary Materials for:

Silva-Junior et al. **Brazilian Amazon Indigenous Territories under deforestation pressure**. *Scientific Reports* (2023).

Table S1. The trend of deforestation increment within indigenous territories between 2013 and 2021 in the Brazilian Amazon Biome.

Indigenous Territories	Sen's Slope (km^2 $year^{-1}$)	p	Mann-Kendall Statistic
Apyterewa	8.582	0.006	26
Cachoeira Seca	6.071	0.013	24
Trincheira Bacaja	4.377	0.006	26
Kayapó	2.838	0.013	24
Munduruku	2.512	0.000	34
Karipuna	1.165	0.021	23
Yanomami	0.921	0.076	18
Uru-Eu-Wau-Wau	0.860	0.180	14
Parque do Xingu	0.441	0.044	20
Sete de Setembro	0.412	0.044	20
Alto Rio Negro	0.382	0.076	18
Andirá-Marau	0.207	0.358	10
Roosevelt	0.180	0.180	14
Maraiwatsede	0.175	0.476	8
Sararé	0.158	0.076	18
Arara do Rio Branco	0.154	0.055	19
Xikrin do Rio Catete	0.147	0.044	20
Araweté	0.147	0.208	13
Kayabi	0.140	0.529	7
Kaxarari	0.126	0.120	16
Amanayé	0.126	0.055	19
Tikuna	0.122	0.612	6
Zoró	0.105	0.612	6
Trombetas/Mapuera	0.098	0.025	22
Vale do Javari	0.095	0.076	18
Waimiri-Atroari	0.093	0.044	20
Ticuna	0.089	0.295	11
Igarapé Lourdes	0.088	0.675	5
Pacaás-Novas	0.084	0.180	14
Apurinã Km 124 BR-317	0.081	0.120	16
Sai-Cinza	0.079	0.260	12

Indigenous Territories	Sen's Slope (km^2 $year^{-1}$)	p	Mann-Kendall Statistic
Boca do Acre	0.077	0.093	17
Mamoadate	0.075	0.476	8
Tenharim Marmelos (Gleba B)	0.074	0.202	13
Coata-Laranjal	0.070	0.059	19
Rio Branco	0.068	0.612	6
WaiApi	0.068	0.287	11
Kanamari do Rio Juru	0.067	0.076	18
Menku	0.065	0.476	8
Sarau	0.065	0.762	4
Tenharim Marmelos	0.064	0.529	7
Baú	0.063	0.093	17
Aripuanã	0.062	0.142	15
Deni	0.062	0.120	16
Terena Gleba Iriri	0.061	0.055	19
Menkragnoti	0.058	0.358	10
Igarapé Lage	0.056	0.476	8
Kaxinawa do Rio Jordão	0.056	0.021	23
Malacacheta	0.052	0.402	9
Cabeceira do Rio Acre	0.047	0.001	30
Koatinemo	0.045	0.089	17
Zuruaha	0.045	0.395	9
Panará	0.042	0.093	17
Tubarão Latunde	0.040	0.073	18
Arara do Igarapé Humaitá	0.033	0.454	8
Kaxinawa da Praia do Carapane	0.032	0.007	26
Igarapé Ribeirão	0.031	0.260	12
WaiWAi	0.031	0.142	15
Kaxinawa Nova Olinda	0.031	0.037	20
Katukina/Kaxinawa	0.031	0.295	11
Parque do Aripuanã	0.029	0.920	2
Peneri/Tacaquiri	0.028	0.461	8
Vui-Uata-In	0.028	0.033	21
Pinatuba	0.028	0.029	20
Rio Gregório	0.028	0.287	11
Nukini	0.027	0.059	19
Caititu	0.024	0.324	10
Betania	0.024	0.078	17
Médio Rio Negro II	0.022	0.138	15
Rio Tea	0.020	0.008	26

Indigenous Territories	Sen's Slope (km^2 $year^{-1}$)	p	Mann-Kendall Statistic
Miratu	0.020	0.675	5
Tabalascada	0.019	0.037	20
Inauini/Teuini	0.018	0.063	17
Cunha-Sapucaia	0.018	0.090	17
Arara	0.018	0.021	23
Kaxinawa do Baixo Rio Jordão	0.017	0.239	12
Jaminaua/Envira	0.015	0.134	14
Paracuhuba	0.015	0.343	10
Vale do Guaporé	0.014	0.529	7
Alto Rio Guamá	0.014	0.540	0
Paquiçamba	0.012	0.358	10
Rio Mequens	0.010	0.461	8
Batovi	0.008	0.508	7
Apurini	0.007	0.232	12
Manoa/Pium	0.006	0.920	2
Parana do Boa Boa	0.005	0.079	15
Capoto/Jarina	0.005	0.752	4
Alto Rio Purus	0.004	0.529	7
Kampa do Rio Amônea	0.004	0.667	5
Balaio	0.003	0.378	9
Erikpatsa	0.003	0.584	6
Tenharim do Igarapé Preto	0.003	0.831	3
Aningal	0.002	0.593	6
Badjônkôre	0.002	0.300	10
Sororó	0.001	0.916	2
Rio Urubu	0.001	0.584	6
Tukuna Umariaeui	0.001	0.584	6
Sararé	0.000	0.043	15
Serra da Moça	0.000	0.053	-16
Jaminawa do Igarapé Preto	0.000	0.082	13
Pium	0.000	0.082	-13
Paumari do Rio Ituxi	0.000	0.101	-14
Sepoti	0.000	0.133	13
Xipaya	0.000	0.133	13
Lago Aiapua	0.000	0.148	-11
Maraã Urubaxi	0.000	0.148	11
Paumari do Lago Manaus	0.000	0.148	11
Pequizal do Naruvôtu	0.000	0.148	-11
Apurini do Igarapé Mucuim	0.000	0.175	8

Indigenous Territories	Sen's Slope (km^2 $year^{-1}$)	p	Mann-Kendall Statistic
Apurini Igarapé Tauamirim	0.000	0.175	8
Barreirinha	0.000	0.175	8
Cajuhiri Atravessado	0.000	0.175	8
Lauro Sodré	0.000	0.175	8
Mapari	0.000	0.175	-8
Nambikwara	0.000	0.175	8
Poyanawa	0.000	0.175	8
São Pedro	0.000	0.175	8
Santa Inez	0.000	0.175	-8
Seruini/Mariene	0.000	0.188	-10
Camicua	0.000	0.210	11
Kaxinawa do Rio Humaitá	0.000	0.210	11
Massaco	0.000	0.210	11
Kulina Igarapé do Pau	0.000	0.316	9
Diahui	0.000	0.333	6
Geralda Toco Preto	0.000	0.333	6
Jabuti	0.000	0.333	-6
Juma	0.000	0.333	6
Kulina do Rio Envira	0.000	0.333	-6
Lago do Beruri	0.000	0.333	6
Mangueira	0.000	0.333	-6
Nova Jacundá	0.000	0.333	-6
Xambio	0.000	0.333	-6
Kampa e Isolados do Rio Envira	0.000	0.353	-9
Kampa do Igarapé Primavera	0.000	0.385	7
Pequizal	0.000	0.385	-7
Campinas/Katukina	0.000	0.420	8
Trincheira	0.000	0.420	8
Jacamim	0.000	0.452	7
Rio Apaporis	0.000	0.452	7
Mawetek	0.000	0.528	6
Arary	0.000	0.561	-4
Lago do Correio	0.000	0.561	4
Paraná do Arauató	0.000	0.561	4
Paumari do Cuniua	0.000	0.561	4
Rio Omerê	0.000	0.561	4
Truaru	0.000	0.561	4
Turé Mariquita II	0.000	0.561	-4
Turé Mariquita	0.000	0.561	4

Indigenous Territories	Sen's Slope (km^2 $year^{-1}$)	p	Mann-Kendall Statistic
Anta	0.000	0.562	-5
Camadeni	0.000	0.562	5
Matintin	0.000	0.562	-5
Rio Jumas	0.000	0.562	-5
Tikuna de Santo Antonio	0.000	0.562	5
Apiaka/Kayabi	0.000	0.565	6
Rio Paru D'este	0.000	0.565	6
Tukuna Porto Espiritual	0.000	0.565	-6
Wawi	0.000	0.565	-6
Jarawara/Jamamadi/Kanamati	0.000	0.616	5
Kaxinawa Seringal Independência	0.000	0.616	5
Zoe	0.000	0.616	5
Nova Esperança do Rio Jandiatuba	0.000	0.705	4
Riozinho do Alto Envira	0.000	0.742	-4
Macarrão	0.000	0.744	4
Acim	0.000	0.772	3
Escondido	0.000	0.772	3
Rio Manicor	0.000	0.772	3
Sucuba	0.000	0.772	-3
Ipixuna	0.000	0.802	3
São Domingos do Jacapari e Estação	0.000	0.802	3
Serra Morena	0.000	0.802	3
Umutina	0.000	0.802	3
Apurini do Igarapé São João	0.000	0.846	2
Kaxinawa Ashaninka do Rio Breu	0.000	0.846	-2
Pirineus de Souza	0.000	0.846	-2
Rio Formoso	0.000	0.846	2
Tapirapé-Karaja	0.000	0.846	-2
Torá	0.000	0.846	2
Igarapé Grande	0.000	0.899	-2
Jaminawa Arara do Rio Bagé	0.000	0.908	-2
Karitiana	0.000	0.908	-2
Sagarana	0.000	0.908	-2
Cuia	0.000	0.912	2
Alto Sepatini	0.000	1.000	-1
Anambé	0.000	1.000	-1
Arara da Volta Grande do Xingu	0.000	1.000	0
Barata Livramento	0.000	1.000	0

Indigenous Territories	Sen's Slope (km^2 $year^{-1}$)	p	Mann-Kendall Statistic
Barreira da Missão	0.000	1.000	-1
Barro Alto	0.000	1.000	0
Cacau do Tarauaca	0.000	1.000	0
Canauanim	0.000	1.000	1
Catipari/Mamoria	0.000	1.000	-1
Fortaleza do Castanho	0.000	1.000	0
Galibi	0.000	1.000	0
Governador	0.000	1.000	0
Igarapé Capana	0.000	1.000	-1
Igarapé do Caucho	0.000	1.000	1
Kuruáya	0.000	1.000	1
Kwazá do Rio São Pedro	0.000	1.000	0
Miguel/Josefa	0.000	1.000	0
Padre	0.000	1.000	0
Parque do Tumucumaque	0.000	1.000	0
Patau/	0.000	1.000	1
Rio Guapor	0.000	1.000	0
Tembé	0.000	1.000	-1
Tikuna de Feijoal	0.000	1.000	0
Tumiã	0.000	1.000	1
Uati-Paraná	0.000	1.000	-1
Nove de Janeiro	-0.001	0.748	-4
Kumaru do Lago Ualá	-0.001	0.584	-6
Estrela da Paz	-0.003	0.834	-3
Trocará	-0.003	0.104	-15
Moskow	-0.004	1.000	-1
Pirahã	-0.008	0.540	0
Nhamundá-Mapuera	-0.012	0.762	-4
Raposa Serra do Sol	-0.013	0.920	-2
Itixi Mitari	-0.014	0.012	-21
Cuiu-Cuiu	-0.015	0.454	-8
Rio Negro Ocaia	-0.015	0.239	-12
Paumari do Lago Marahã	-0.019	0.114	-16
Raimundo	-0.020	0.084	-16
Boqueirão	-0.023	0.134	-14
Arara	-0.023	0.103	-16
Kulina do Medio Jurua	-0.031	0.402	-9
Gavino	-0.033	0.055	-19
Rio Biá	-0.033	0.171	-14

**Indigenous Territories**	**Sen's Slope (km^2 $year^{-1}$)**	**p**	**Mann-Kendall Statistic**
Arariboia	-0.050	0.762	-4
Parakanã	-0.066	0.012	-25
Uati parano	-0.150	0.120	-16
Caru	-0.216	0.006	-26
Urubu Branco	-0.218	0.180	-14
Awa	-0.624	0.021	-23
Alto Turiaçu	-0.773	0.044	-20

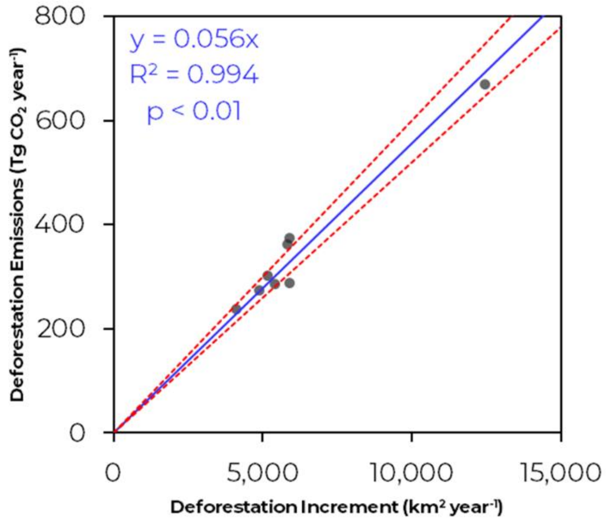

Figure S1. The linear regression model between the annual increment of deforestation in the Brazilian Amazon biome and its carbon dioxide (CO_2) emissions. The linear regression line is drawn in blue, and the red dashed lines are the 95% confidence interval. The raw values of deforestation and CO_2 emissions used in the linear regression are available in Table S2 below. The figure was elaborated by Celso H. L. Silva-Junior using the software Microsoft Office Excel (https://www.microsoft.com/pt-br/microsoft-365).

Table S2. The raw values of deforestation and CO_2 emissions used in the linear regression of Figure S1.

Year	Deforestation (km^2 year^{-1})	CO_2 Emission (Tg year^{-1})
2008	12,445	669
2009	5,902	373
2010	5,843	363
2011	5,400	286
2012	4,128	237
2013	5,153	302
2014	4,874	274
2015	5,907	288
